# Centrosomal and mitotic abnormalities in cell lines derived from papillary thyroid cancer harboring specific gene alterations

**DOI:** 10.1186/1755-8166-4-26

**Published:** 2011-11-16

**Authors:** Irena Maric, Silvia Viaggi, Paola Caria, Daniela V Frau, Paolo Degan, Roberta Vanni

**Affiliations:** 1 Dipartimento per lo Studio del Territorio e delle sue Risorse, Università degli Studi di Cagliari, Genova, 16132, Italy; 2 IRCCS Azienda Ospedaliera Universitaria San Martino - IST - Istituto Nazionale per la Ricerca sul Cancro, Genova, 16132, Italy; 3 Dipartimento di Scienze e Tecnologie Biomediche, Università di Cagliari, 09042, Italy

**Keywords:** thyroid carcinoma, centrosome, mitotic spindle, *RET/PTC*, *BRAF*

## Abstract

**Background:**

Differentiated thyroid carcinoma offers a good model to investigate the possible correlation between specific gene mutations and chromosome instability. Papillary thyroid neoplasms are characterized by different mutually exclusive genetic alterations, some of which are associated with aneuploidy and aggressive phenotype.

**Results:**

We investigated the centrosome status and mitotic abnormalities in three thyroid carcinoma-derived cell lines, each maintaining the specific, biologically relevant gene alteration harbored by the parental tumors: *RET/PTC1 *rearrangement in TPC1; heterozygous and homozygous *BRAF^V600E ^*mutation in K1 and in B-CPAP, respectively. B-CPAP cells showed a statistically significant (*P *< 0.01) higher frequency of abnormal mitotic figures compared to TPC1 and K1 cells.

**Conclusions:**

Our data indicate that *RET/PTC1 *oncogenic activity is not related to mitotic chromosome impairment and missegregation whereas, based on the consistent difference in types/frequencies of centrosome and spindle abnormalities observed between K1 and B-CPAP cells, the hetero/homozygous allelic status of *BRAF^V600E ^*mutation seems to be not irrelevant in respect to chromosomal instability development.

## Background

Chromosomal instability (CIN), a genetic condition that promotes a high rate of chromosome missegregation during mitosis, is a shared feature of most cancers. Aneuploidy is a distinctive trait of most human cancers and has been linked to high tumour grade, advanced stage, and poor prognosis; however, its role in neoplastic transformation and the relationship with CIN are somewhat unclear [1]. A variety of alterations have been proposed as being responsible for CIN, including defects in the spindle checkpoint, defective sister-chromatid cohesion, kinetochore assembly, upregulation of cyclins, erosion of telomeres and centrosome abnormalities [2]. The normal function and numeral integrity of centrosomes can be affected directly by the mutated products of certain proto-oncogenes and tumour-suppressor genes [3,4]. In particular, centrosome amplification (the presence in the cell of more than two centrosomes) seems to be an early event in tumourigenesis [5], and it may affect CIN in different ways, depending on the capacity to maintain a correct bipolar chromosome segregation in the presence of multipolar spindles. In addition to amplification, structural and functional defects of centrosomes may influence CIN [4].

Papillary thyroid carcinoma (PTC), accounting for approximately 80% of differentiated thyroid carcinoma (DTC), has a number of variants with specific histological characteristics. The classical form of PTC may show activation of the *BRAF *gene or *RET/PTC *variants, in 40-45% and 10-20% of cases, respectively [6]. The two alterations are usually mutually exclusive. In different PTC-derived cell lines, the alternative presence of either *BRAF *mutation or *RET/PTC *rearrangement has been confirmed [7], reinforcing the hypothesis that *RET *and *BRAF *changes are alternative oncogenic events. The majority of papillary thyroid carcinoma shows stable karyotypes, including the cases in which aneuploid karyotypes have been described [8], being chromosomal instability only observed in the more advanced clinicopathological stages [9]. Intriguingly, however, it has been demonstrated that conditional *BRAF^V600E ^*expression induces chromosomal instability in thyroid PCCL3 cells [10], suggesting a possible key role of the gene in determining CIN. Moreover, the contribution of centrosome in the morphogenetic process of PTC nuclear changes has been recently suggested [11]. Nevertheless, little is known about the relationship among PTC molecular events, aneuploidy and genomic instability of these tumours.

TPC1, K1 and B-CPAP PTC-derived cell lines are characterized by the specific genetic alterations of the parental tumours from which they have been derived, namely *RET/PTC1 *rearrangement in TPC1 [12], *BRAF^V600E ^*mutation in K1 and in B-CPAP [7]. To investigate the possible relationship between the relevant specific gene alterations and mitotic behavior of thyroid cancer cells, we studied centrosome abnormalities and mitotic spindle aberrations of these three human PTC-derived cell lines and of NTHY-ORI3.1 cells derived from human follicular epithelial cells.

## Results

### Fluorescence in situ hybridization

*RET *FISH pattern intepretation. Diploid cells that have the chromosomal rearrangement involving the *RET *gene will show a discrete red signal distanced from a discrete green signal, indicating the breakage of one *RET *allele, while the second allele will show a red/green fused signal. Depending on chromosome 10 polysomy, this pattern will change accordingly.

### NTHY-ORI-3.1 cell line

**Figure 1:**
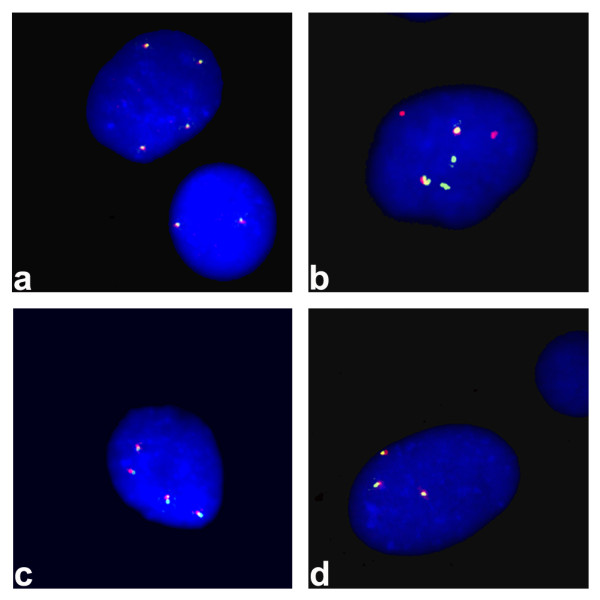
**Fluorescence in situ hybridization**. FISH with a home-brew dual color break-apart probe set for *RET *gene (5' labelled with Spectrum Orange and 3' labeled with Spectrum Green). Cells with unrearranged *RET *show intact red/green signals (one fused signal for each *RET *gene); cells with rearranged *RET *show probe signal split into its 5' (red signal) and 3' (green signal) portions (two discrete red and green signals for each rearranged *RET *gene). NTHY-ORI 3-1 representative nuclei with four (a, top) and two (a, bottom) copies of intact *RET*; *TPC1 *representative nucleus with two copies of intact *RET *(two red/green signals) and two copies of rearranged *RET *(two discrete red and green signals) (b); K1 representative nucleus with four copies of intact *RET *(c); B-CPAP repesentative nucleus with three copies of intact *RET *(d). Nuclei are counterstained with 4',6-diamidino-2-phenylindole.

Nuclei of the NTHY-ORI-3.1 cell line showed two, three or four red/green intact signals, indicating no *RET *rearrangement and the presence of tri-tetraploid clones, in addition to the diploid one (Figure 1a).

### PTC-derived cell lines

Most nuclei of the TPC1 cell line showed two intact red/green signals and two red and two green discrete signals, indicating the presence of *RET *breakage in the tetraploid clone (Figure 1b). A minor diploid clone showed one intact red/green signal and one red and one green discrete signals. Nuclei of the K1 cell line showed four intact red/green fluorescent signals, as expected for tetrasomy 10 (Figure 1c) in a tetraploid cell line. Nuclei of the B-CPAP cell line showed red/green intact signals. According to the presence of a major clone with trisomy 10, most of the nuclei showed three intact red/green signals (Figure 1d).

These results indicated that only TPC1 cells had *RET *rearrangement.

### Immunofluorescence results

### NTHY-ORI-3.1 cell line

**Figure 2:**
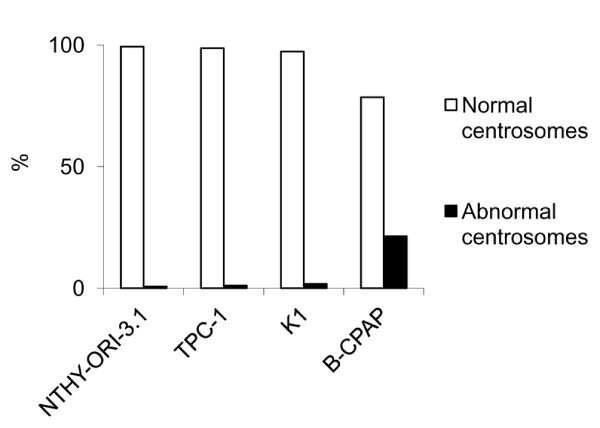
**Centrosome analysis (immunofluorescence)**. Distribution of centrosome abnormalities in the studied cell lines: preponderance of abnormal centrosomes in B-CPAP cells.

**Figure 3:**
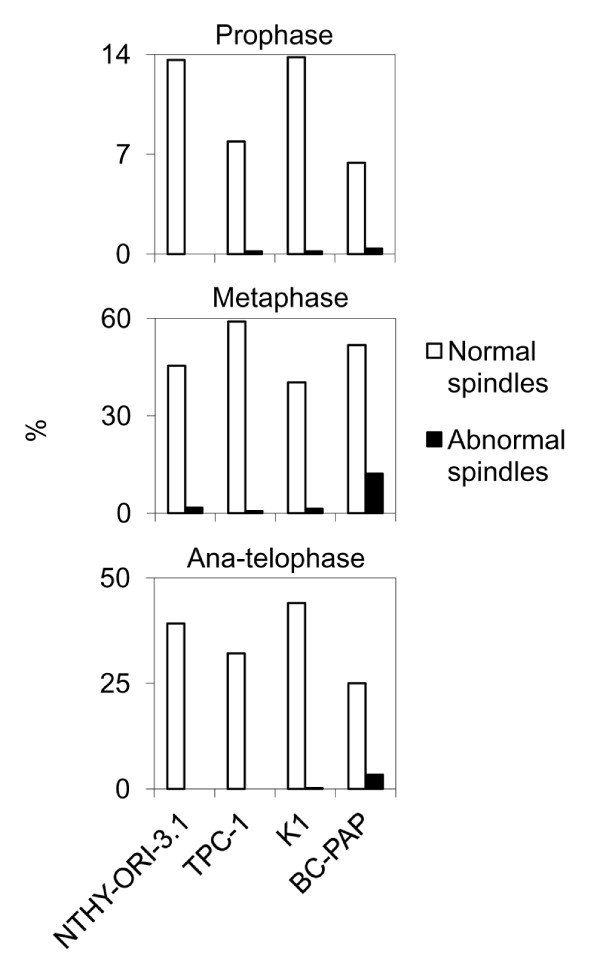
**Mitotic spindle analysis (immunofluorescence)**. Distribution of spindle mitotic abnormalities in the studied cell lines: preponderance of metaphase spindle abnormalities in B-CPAP cells.

A very low frequency of centrosome amplification (0,7%) and spindle abnormalities (1,7%) were observed in the control SV-40-immortalized cell line NTHY-ORI-3, as an effect of SV40 immortalization (Figures 2 and 3).

### PTC-derived cell lines

**Figure 4:**
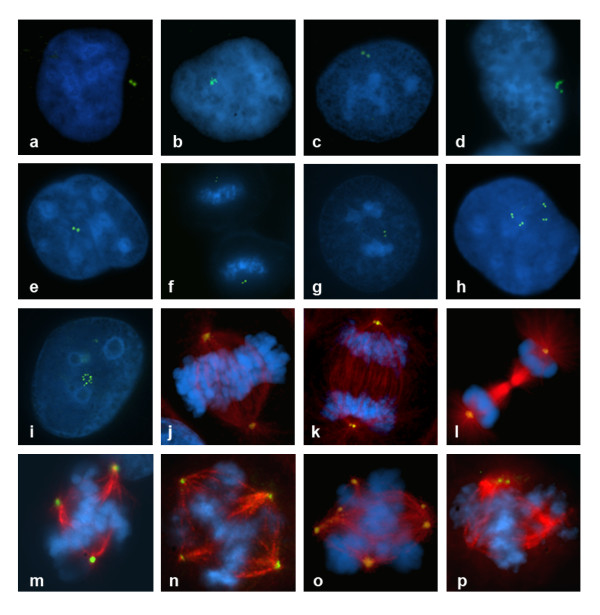
**Centrosome and mitotic spindle immunofluorescence, representative images**. Centrosome γ-tubulin immunostaining (green signal): normal centrosome in NTHY-ORI 3-1 (a), TPC-1 (b) and K1 (c) cell lines; amplified centrosome in B-CPAP cell line (d). Centriol centrin 2 immunostaining (green signal): one pair of centriols in NTHY-ORI 3-1 (e), TPC-1 (f) and K1 (g) cell lines; amplification in B-CPAP cell line: four pairs (h) and five pairs (i) of centriols. Gamma- and β-tubulin immunostaining highlighting centrosomes (green signal) and mitotic spindle microtubules (red signal): normal mitotic figures in NTHY-ORI 3-1 (j), TPC-1 (k), and K1 (l) cell lines; abnormal mitotic figures in B-CPAP cell line: a tripolar metaphase (m), a quadripolar metaphase (n); a multipolar metaphase (o), and a tripolar metaphase with two acentrosomal spindle poles (p).

B-CPAP cells displayed a significantly higher (*P *< 0.01) frequency of centrosome amplification (21,4%), when compared to normal thyroid tissue derived NTHY-ORI-3.1 cell line (0,7%) and the papillary thyroid tumour derived TPC1 (1,1%) and K1 (1,8%) cell lines (Figure 2). In detail, NTHY-ORI-3.1 cells showed normal centrosomes (Figure 4a) as well as TPC1 (Figure 4b) and K1 (Figure 4c) cells, whereas in B-CPAP cells centrosome amplification was observed (Figure 4d). The immunofluorescence with anti-centrin 2 antibody showed one or two centriolar pairs in NTHY-ORI-3.1 (Figure 4e), TPC1 (Figure 4f), and K1 (Figure 4g), and amplification (centriolar pairs > 2) in B-CPAP (Figure 4h, i).

Among the four analyzed cell lines (Figure 3, Figure 4j-p), only B-CPAP cells showed a statistically significant (*P *< 0.01) higher frequency of abnormal mitotic figures (15.9%) (Figure 4m, n, o) compared to both follicular cells derived NTHY-ORI-3.1 (1,7%) and tumour derived TPC1 (0,9%) and K1 (1,8%) cell lines. A small fraction of B-CPAP cells showed acentrosomal spindle poles (Figure 4p).

### Giemsa-staining results

### NTHY-ORI-3.1 cell line

**Figure 5:**
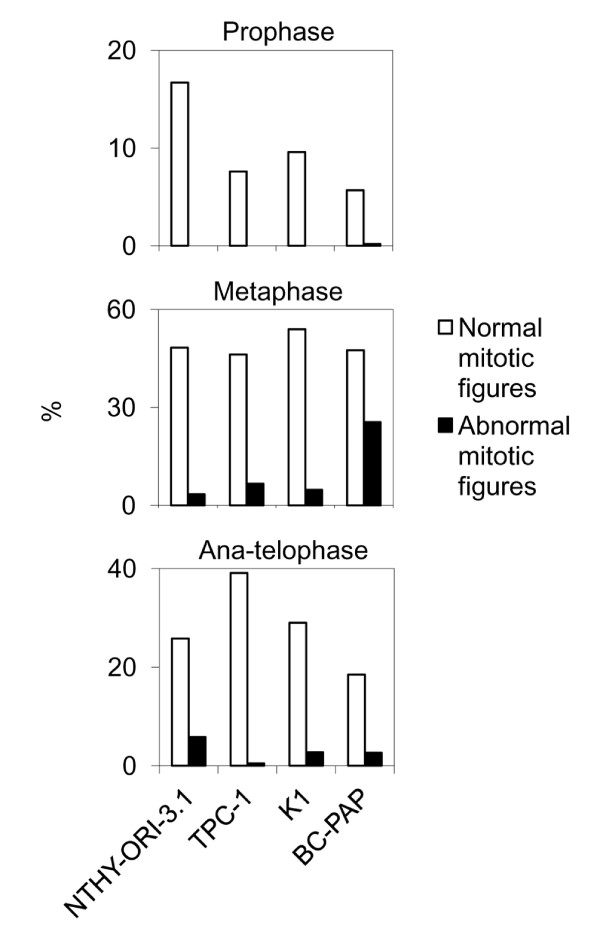
**Mitotic figures analysis (Giemsa staining)**. Distribution of abnormal mitotic figures in the studied cell lines: preponderance of abnormal metaphases in B-CPAP cells, and difference of mitotic alterations between the TPC1 and K1 small abnormal cell populations (K1 has preponderance of abnormal ana-telophase).

**Figure 6:**
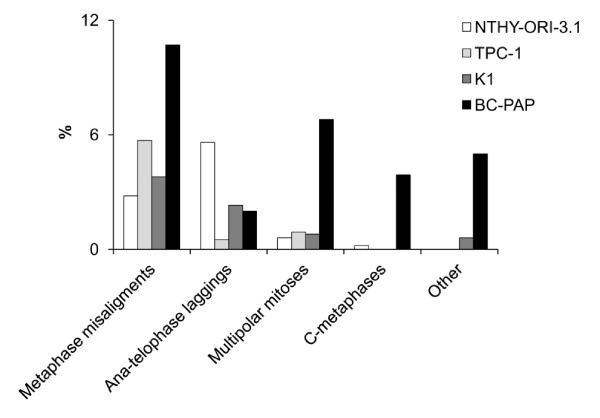
**Distribution of abnormal figures during mitotic phases (Giemsa staining)**. Overall distribution of different classes of aberrations during mitosis in the studied cell lines: among the PTC-derived cell lines, B-CPAP shows the highly consistent number of mitotic abnormal figures in all phases. Ana-telophase laggings in NTHY-ORI 3-1 cells are possibly a consequence of SV40-transfection.

NTHY-ORI-3.1 cells showed a total of 9.2% aberrant mitotic figures, mainly consisting of metaphases with misaligned chromosomes (2,8%) and anaphases with chromosome laggings (5,6%). A very low percentage of mitosis (0,6%) showed multipolar spindles (Figures 5 and 6).

### PTC-derived cell lines

**Figure 7:**
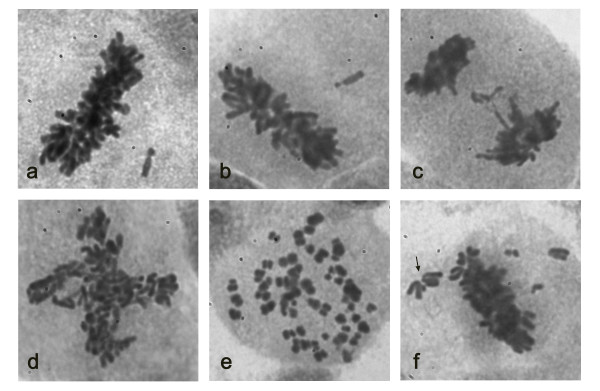
**Abnormal mitotic figures, Giemsa staining representative images**. TPC1, metaphase displacement (a); B-CPAP, metaphase displacement (b); B-CPAP, anaphase lagging (c); B-CPAP, quadripolar metaphase (d); B-CPAP, c-metaphase (e); B-CPAP, chromosome displacements suggesting the presence of an extra centrosome: the angle formed by the chromosomes on the right (arrow) possibly indicates that they are attached to microtubules from an extra centrosome (f).

Among the three thyroid cancer derived cell lines, both TPC1 and K1 cells showed approximately 7% aberrant mitotic figures (Figures 5 and 6). Most of the TPC1 abnormal mitosis were metaphases with misaligned chromosomes (Figure 7a) (5,7%) and a minority involved chromosome laggings (0,5%) and multipolar mitoses (0,9%). The K1 cells showed metaphases with misaligned chromosomes (3,8%) or chromosome laggings (2,3%), and few multipolar mitosis (0,8%). Comparing the abnormal mitotic figures of these cell lines with those of Giemsa-stained B-CPAP cells, a statistically significant (*P *< 0.01) higher frequency of abnormal mitotic figures was found in B-CPAP cells (28.3%) (Figures 4 and 5), including misaligned chromosomes (10,7%) (Figure 7b), ana-telophase lagging chromosomes (2,0%) (Figure 7c), multipolar figures (6,8%) (Figure 7d), c-metaphases (3,8%) (Figure 7e), and other alterations (5%), such as highly polyploid figures, pulverized metaphases and others that were difficult to classify (Figure 7f).

## Discussion

A number of gene alterations, such as point mutations in *RAS *and *BRAF genes*, point mutations or amplification of *PIK3CA*, and fusion genes involving *RET, NTRK1 *and *PPARγ *are known to frequently occur in differentiated thyroid carcinoma [6,13], and are correlated to different morphological subtypes. Moreover, they are characterized by different chromosome patterns, defining specific cytogenetic subgroups which are often correlated with different histopathological features [14].

Common mutations found in the papillary histologic subtype are point mutations of the *BRAF *and *RAS *genes as well as *RET/PTC *rearrangements, which are considered molecular markers of diagnostic and prognostic significance.

TPC1, K1, and B-CPAP are among the most frequently used papillary thyroid carcinoma-derived cell lines for *in vitro *investigations of thyroid oncogenesis. Although tumour derived cell lines are considered to be the outcome of adaptation and *in vitro *evolution leading to a common undifferentiated phenotype [15], all these cell lines maintained the biologically relevant oncogenic events specific to the different subtypes of their parental PTCs. TPC1 and K1 cell lines, bearing *RET/PTC1 *and heterozygous *BRAF^V600E ^*mutation respectively, had an extremely low number of aberrant cells. No statistically significant differences were found in centrosome and spindle alterations, as well as multipolar mitoses, compared to follicular thyroid cell-derived NTHY-ORI-3.1 cell line, which indeed is a SV-40 immortalized cell line and a population of abnormal mitotic cells can be expected. In contrast, the B-CPAP cell line, bearing a homozygous *BRAF^V600E ^*mutation, showed remarkable alterations: several mitotic abnormalities such as metaphase chromosome misalignments, multipolar figures, and c-metaphases were observed. Intriguingly, these types of alterations were barely observed in the K1 cell line harbouring heterozygous *BRAF^V600E ^*mutation. The consistent difference in types/frequencies of centrosome and spindle abnormalities observed between K1 and B-CPAP cells might suggest that the allelic status of *BRAF^V600E ^*mutation is not irrelevant in respect to CIN development, and that the hetero/homozygous mutational status of the gene may influence at different rate the onset of aneuploidy in PTC cells. B-CPAP cells show a mitotic machinery deeply impaired, with centrosome amplification, acentrosomal spindle poles and chromosome misalignment, giving rise to an increased mitotic instability, whereas *BRAF^V600E ^*heterozygous K1 cells maintain correct centrosome features and spindle polarity in the majority of cells. In fact, B-CPAP cell line has a less stable karyotype compared to K1. Moreover, as expected, because of centrosome amplification, B-CPAP showed a significantly higher frequency of multipolar mitoses compared to K1. A small proportion of these mitoses showed acentrosomal spindle poles, which are reminiscent of spindle formation involving nucleation through a chromatin-dependent spindle assembly pathway [16]. Very recently, a link between the *BRAF^V600E ^*oncogene and chromosome instability in melanoma has been suggested [17]. More than 65% of cutaneous melanoma [18] and approximately 45% of PTC [6] share the same *BRAF^V600E ^*mutation, however, differently from melanoma in which aneuploid karyotypes are frequent [8] and homozygous *BRAF^V600E ^*mutation is described [19], PTC usually has heterozygous mutation [20]. To the best of our knowledge, *in vivo *homozygous *BRAF^V600E ^*mutation in thyroid tumours has not been yet described, and it is likely that homozygous cell lines have acquired the second mutation during their establishment. Nevertheless, as B-CPAP is the only of the three PTC-derived cell lines harboring a mutation in *TP53*, which has been closely related to centrosome duplication and genomic integrity maintenance [4,21], the possibility that *TP53 *mutation could be involved in the observed enhanced instability, can not be ruled out.

Chromosome number in K1 and TPC1 cell lines is maintained rather stable in culture. However, K1 cells karyotype is characterized by both structural and numerical chromosome changes, whereas TPC1 karyotype is characterized by structural changes [15]. Indeed, the small population of K1 cells bearing mitotic abnormalities shows mainly ana-telophase laggings, compared to the small population of mitotic abnormal TPC1 cells (P < 0.05). As lagging chromosomes at anaphase represent a potential source of aneuploidy, the presence of this small abnormal ana-telophase population may explain the presence of clonal chromosome numerical changes in K1 karyotype. Intriguingly, *BRAF^V600E ^*positive PTC may harbour aneuploidy [9], whereas *RET/PTC *positive carcinoma very rarely shows numerical chromosome changes - and indeed *RET/PTC *rearrangements have been reported in non-malignant thyreocytes [22]. A direct ascertainment of the mitotic behavior and centrosome status of PTC with *BRAF^V600E ^*mutation in primary PTCs might favourably confirm this view.

## Conclusions

Our data suggest that difference in spindle abnormalities and possibly in centrosome amplification may depend on the *BRAF^V600E ^*heterozygous/homozygous mutational status. Whether the proneness to accumulate numerical chromosome changes only in the more rare advanced forms of PTC might depend on the gain of a second *BRAF^V600E ^*mutation, and/or accumulation of other gene alterations, remains to be clarify.

The degree and type of genetic instability in cancer is emerging as an important feature, also considered as a possible ancillary and integrative parameter in tumour classification. Besides the prognostic relevance of chromosomal instability, CIN could also contribute to the ability of cancer cells to acquire chemoresistance, generating occasionally cells with the capacity to grow more efficiently in adverse environments. In this view, investigation of the correlation between neoplasia gene-specific mutational status and chromosomal instability could provide better targets for gene-specific therapies.

## Methods

### Cell lines

The PTC-derived TPC1 and B-CPAP cell lines were kindly provided by Dr. Fusco (Medical School, University Federico II of Naples, Naples, Italy), the PTC-derived K1 cell line was purchased from Health Protection Agency Culture Collections [23]. TPC1, K1 and B-CPAP cell lines were chosen according to their reported molecular and cytogenetic features: the TPC1 cell line originally showed a near-diploid karyotype and contains a *RET/PTC1 *rearrangement [7], the B-CPAP cell line has an aneuploid karyotype (chromosome mode 72) [24] and *BRAF^V600E ^*homozygous mutation [7], the K1 cell line had a near tetraploid karyotype [25] and *BRAF^V600E ^*heterozygous mutation [7]. We confirmed by FISH that the *RET *rearrangement was present only in TPC1 cells. TaqMan Real-Time polymerase chain reaction assay confirmed the presence of the *BRAF^V600E ^*mutation in B-CPAP and K1 cells. Karyotyping revealed tetraploidization of TPC1 (a phenomenon already reported in this cell line) [25] and maintenance of the described B-CPAP [23] and K1 [25] chromosome patterns. K1 cells are also characterized by *PIK3CA *amplification [26], which is absent in TPC1, B-CPAP, and NTHY-ORI 3-1 cell lines. For comparison, a commercially available [23] NTHY-ORI 3-1 cell line was used [27]. This SV40-transfected cell line, obtained from human normal thyreocytes, bears wild-type *RET *and *BRAF *genes and retains some morphologic and physiological characteristics of normal thyreocytes, without evidence of malignant transformation [27]. *TP53 *mutation was present in B-CPAP [7] cells. All cell lines lack *RAS *mutations [7]. The cell lines were maintained in DMEM/F12 (Sigma-Aldrich, Milan, Italy) [except NTHY-ORI 3-1 that was grown in RPMI 1640 (Gibco-BRL. Life Technologies, Milan. Italy)] supplemented with 10% fetal bovine serum (Gibco-BRL), at 37°C in humidified 5% CO2.

### Fluorescence in situ hybridization

To investigate the presence of *RET/PTC *rearrangement, double target dual color FISH (i.e. the simultaneous hybridization of two probe sets labelled with different fluorophores) was performed using two 3' and two 5' bacterial artificial chromosome (BAC) clones flanking the common *RET *breakpoint. 5' BAC clone RP11-686A03 and RP11-290I03 (CHORI, Oakland, CA, U.S.A.) were directly labelled with Spectrum Orange fluorophore-conjugated dUTP (Abbott Molecular/Vysis, Downers Grove, IL) and 3' BAC clones RP11-818P01 and RP11-696N03 (CHORI), were labelled with Spectrum Green (Abbott Molecular/Vysis). 20 ng/μl of each probe set were simultaneously hybridized on nuclei: denaturation 5 minutes at 75°C, hybridization 16 h at 37°C in Hybrite™ (Abbott Molecular/Vysis). Slides were counterstained with antifading solution (200 ng/ml 4',6-Diamidino-2-phenylindole, DAPI) (Sigma-Aldrich). Hybridization signals were evaluated by scoring 200 interphase nuclei for each cell line, using a digital image analysis system based on an epifluorescence Olympus BX41 microscope and charge-coupled device camera (Cohu), interfaced with the CytoVysion system (software 3.93.2 Applied Imaging, Pittsburg, PA, USA). The Spectrum Orange, Spectrum Green, and DAPI images were acquired with selective single-bandpass filters at 1000× optical magnification.

### Immunofluorescence staining

Cells for immunofluorescence were cultured directly on flaskette glass slides (NUNC A/S, Roskilde, Denmark). The cells were fixed with methanol:acetone (1:1) for 10 min at -20°C, followed by 10 min methanol at -20°C. Centrosomes and mitotic spindle were labeled with a rabbit polyclonal anti-γ-tubulin antibody (Sigma-Aldrich) and with a mouse anti-β-tubulin antibody (Sigma-Aldrich), respectively. After 1 h incubation with the antibodies diluted 1:200 in PBS/1% BSA at 37°C and 3 × 5 min washing in PBS, the slides were incubated with fluorescein isothiocyanate (FITC)-coupled anti-rabbit secondary antibody (Sigma-Aldrich) and TRITC-conjugated anti-mouse secondary antibody (Sigma-Aldrich) for 30 min at 37°C. After 3 × 5 min washing in PBS, the cells were counterstained with DAPI. Centrosome structural abnormalities were considered to be present when a diameter of at least twice that of normal centrosomes in lymphocytes and in NTHY-ORI 3-1 cells was observed; amplification was recorded when there were more than two centrosomes per cell [28]. At least 200 consecutive cells per sample were examined. Centrioles were labeled with a rabbit anti-centrin 2 antibody (dilution 1:200) (Santa Cruz Biotechnology, Santa Cruz (CA), USA), applying the same procedure described above. Preparations were observed using an epifluorescence microscope (Provis AX70, Olympus, Milan, Italy), and images were acquired with a digital CCD camera (C4742 Orca II, Hamamatsu, Japan) driven by Cytovision (Applied Imaging Corp., Santa Clara, CA, USA). The DAPI, FITC and TRITC images were acquired with selective single-bandpass filters at 1000× optical magnification.

### Giemsa staining

The cells grown on slides were washed twice with PBS, fixed with methanol:acetic acid (3:1) for 30 min at 4°C, and stained in 4% buffered Giemsa stain (pH 7.2) for 25 min. At least 200 consecutive mitotic figures per sample were examined by light microscopy, and images were acquired with a CCD camera at 1000× optical magnification.

### Statistics

Frequency distributions of all variables were calculated, and chi-square analyses were used for categorical comparisons. A p-value < 0.01 was considered statistically significant.

## List of abbreviations

(BAC): Bacterial artificial chromosome; (CIN): Chromosomal instability; (DAPI): 4',6-Diamidino-2-phenylindole; (DTC): Differentiated thyroid carcinoma; (FISH): Fluorescence in situ hybridization; (PTC): Papillary thyroid carcinoma.

## Competing interests

The authors declare that they have no competing interests.

## Authors' contributions

IM and PD carried out the immunofluorescence studies, PC and DVF carried out the conventional and molecular cytogenetic studies and performed the statistical analysis. SV and RV are the project coordinators and designed the study. IM participated in the study design. All authors participated to the first draft of the manuscript and read and approved the final manuscript.
